# Hall of Fame among Pro-inflammatory Cytokines: Interleukin-6 Gene and Its Transcriptional Regulation Mechanisms

**DOI:** 10.3389/fimmu.2016.00604

**Published:** 2016-12-19

**Authors:** Yang Luo, Song Guo Zheng

**Affiliations:** ^1^Department of Clinical Immunology of the Third Affiliated Hospital at the Sun Yat-sen University, Guangzhou, China; ^2^Division of Rheumatology, Department of Medicine at Penn State Hershey College of Medicine, Hershey, PA, USA

**Keywords:** interleukin-6, interleukin-6 gene, pro-inflammatory cytokines, transcriptional regulation, signaling pathway

## Abstract

Pro-inflammatory cytokines that are generated by immune system cells and mediate many kinds of immune responses are kinds of endogenous polypeptides. They are also the effectors of the autoimmune system. It is generally accepted that interleukin (IL)-4, IL-6, IL-9, IL-17, and tumor necrosis factor-α are pro-inflammatory cytokines; however, IL-6 becomes a protagonist among them since it predominately induces pro-inflammatory signaling and regulates massive cellular processes. It has been ascertained that IL-6 is associated with a large number of diseases with inflammatory background, such as anemia of chronic diseases, angiogenesis acute-phase response, bone metabolism, cartilage metabolism, and multiple cancers. Despite great progress in the relative field, the targeted regulation of IL-6 response for therapeutic benefits remains incompletely to be understood. Therefore, it is conceivable that understanding mechanisms of IL-6 from the perspective of gene regulation can better facilitate to determine the pathogenesis of the disease, providing more solid scientific basis for clinical treatment translation. In this review, we summarize the candidate genes that have been implicated in clinical target therapy from the perspective of gene transcription regulation.

## Introduction

Inflammation is beneficial for pathogen clearance and protection against infection; therefore, pro-inflammatory cytokines are regulators of host responses to infection, inflammation, and trauma, which can also make disease worse in pathological conditions (1, 2). These cytokines at least include interleukin (IL)-1, tumor necrosis factor (TNF), interferon (IFN)-γ, and the IL-6, which is the focus of this review (3). Albeit their biological activities widely overlap, each of them has its own biological properties (4–6). IL-6, which was first identified as an antigen non-specific B-cell differentiation factor, was then named as B-cell stimulatory factor 2. It is a glycoprotein with a molecular weight of 26 kDa (7, 8). Human IL-6 consists of 184 amino acids with 2 potential *N*-glycosylation sites and four cysteine residues (9).

## IL-6 Receptor and Its Signaling Pathway

**Figure 1:**
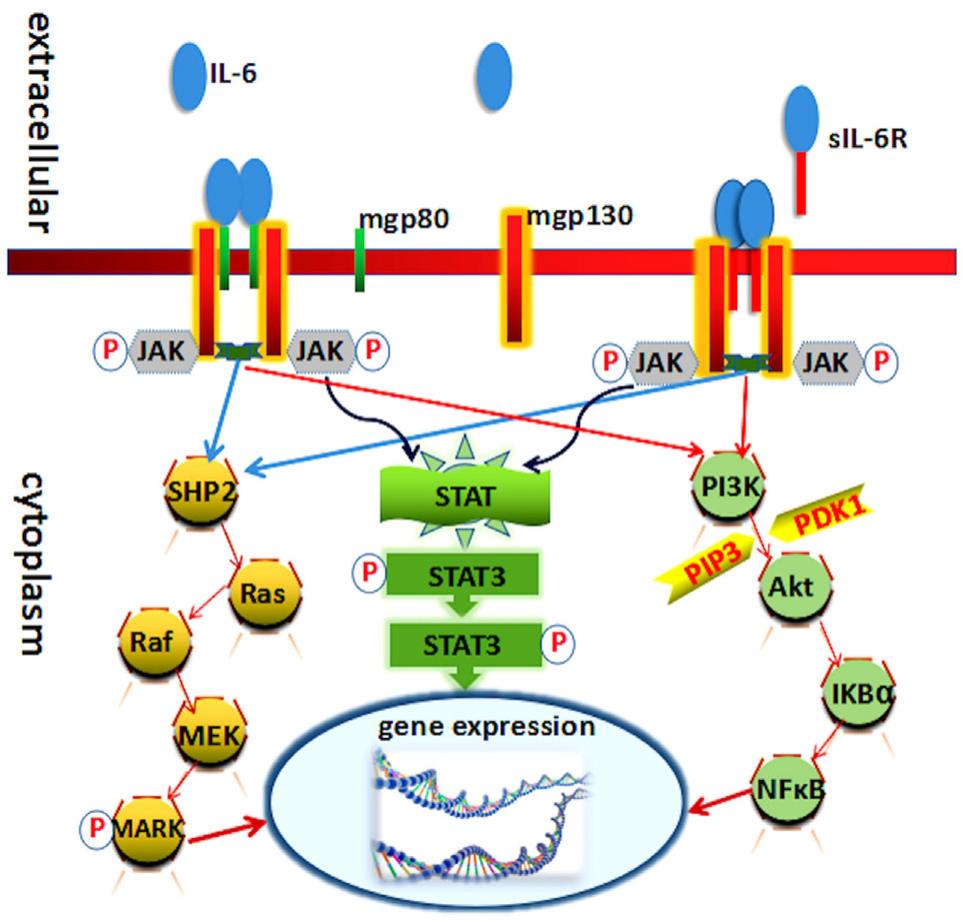
**Known molecules involve in interleukin (IL)-6 signal pathway cascades**. Schematic representation of the functional organization of IL-6 receptor and its three downstream transduction. IL-6 cytokine yields its biological effects *via* two receptors: mgp130 (membrane-bound gp130) and mgp80 (membrane-bound gp80). Each receptor can interact with Janus kinase (JAK) directly. The three pathways all needed JAK and its phosphorylation.

Interleukin-6 exerts its activity mainly through binding to the cell membrane IL-6 receptor (IL-6R). Cell membrane IL-6R consists of two subunits, IL-6Rα (gp80 or CD126), a 80-kDa type I transmembrane protein, and IL-6Rβ (gp130 or CD130), a 130-kDa second signal transmembrane protein. The soluble IL-6R (sIL-6R), which is cleaved from the cell membrane, can still bind its ligand IL-6 (10–12). The paradigm of IL-6 signal transduction *via* the membrane bound IL-6R is called “classic signaling.” Conversely, when it signal goes through sIL-6R, it is called “trans-signaling” (13–15). Generally, IL-6 binding with gp80 and gp130 carries on the conduction of biological signal through three pathways (Figure 1).

Interleukin-6 receptor α (gp80) is mainly expressed on immune cells and therefore immune responses. Recent studies have demonstrated that IL-6 affects the development and balance of Th17 and regulatory T cells, being responsible for the consequence of inflammatory diseases (16). IL-6Rβ is expressed by various cells types, such as lymphocyte, neutrophils, monocytes, macrophages, hepatocytes, affecting immune systems, and others (17, 18).

## JAK/STAT Pathway

Interleukin-6 and IL-6R binding initiate the activation of Janus kinase (JAK), one of the tyrosine kinase family members. The activation of these kinases in turn leads to tyrosine phosphorylation and activation of signal transducer and activator of transcription (STAT3) (19, 20). Phosphorylation and activation of these kinases induced by heterodimer/homodimer gp130:gp130 or gp130:leukemia inhibitory factor receptor (ILFR) result in the phosphorylation of six tyrosine residues on the gp130 and ILFR. Following phosphorylation, a variety of molecules at the SH-2 domain were upregulated, such as SHP-2, Shc, and STATs. STAT3 then forms a dimer to transmit signals from the cell membrane to the nucleus (19, 21). The IL-6/JAK/STAT3 canonical pathway regulates the expression of several genes leading to the induction of cell growth differentiation and survival (22).

## Ras/Mitogen-Activated Protein Kinases (MAPK) Pathway

Ras protein is also activated in response to IL-6 that involves in the formation and the activation of complex compounds:Grb2 (growth factor receptor-binding protein) and Shc (SH2 and collagen homology domain containing protein) (23). Subsequently, it activates downstream signaling of MAPK and leads to an increase in its serine/threonine kinase activity. Several substrates involve in the MAPK phosphorylation actions, like c-Myc, c-Jun, and c-Fos. It mediates diverse effects including cell growth stimulation acute-phase protein synthesis and immunoglobulin synthesis (24, 25).

## Phosphoinositol-3 Kinase (PI3K)/Akt Pathway

The PI3K–protein kinase B (PkB)/Akt pathway also plays an indispensable role in the transduction of IL-6 signal, especially in the antiapoptotic effect of IL-6 in prostate cancer cells. PI3K protein modifies certain phosphatidylinositides in phosphorylate phosphatidylinositol-4,5-bisphosphate to phosphatidylinositol-3,4,5-trisphosphate (PIP3). PIP3 in turn phosphorylates and activates serine/threonine kinase PkB/Akt which is recruited to the plasma membrane. Akt can be activated by phosphoinositide-dependent kinase-1 through phosphorylation and then phosphorylates several downstream targets to upregulate cellular survival signaling pathways (24, 26).

## Cis-Acting Element

**Figure 2:**
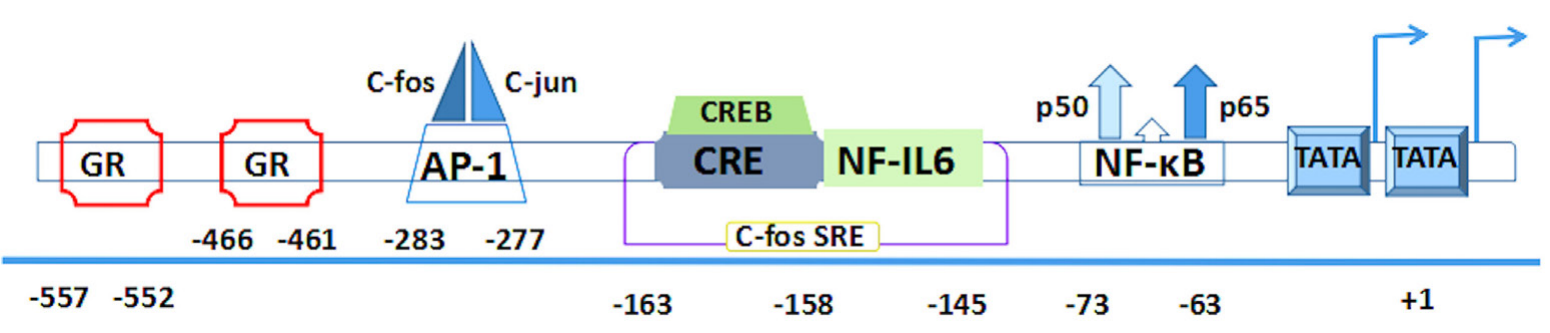
**The human interleukin-6 gene prompter with putative cis-regulatory elements and approximate binding site relative to trans-regulatory factors**.

The corresponding human gene of IL-6 including three transcription start sites and three TATA like sequences (TATA boxes) is localized on chromosome 7p21 and consists of five exons and four introns (27). Several of its cis-acting elements are located in a 1.2-kb fragment of the 5′-flanking region (28, 29) (Figure 2).

First two pairs of glucocorticoid response elements (GREs) are located at the positions –557 to –552 and –466 to –461 in the human IL-6 gene (28). Then comes the activator protein 1 (AP-1)-binding site. It is a consensus sequence (*TGAGTCA*) found at the position –283 to –277 (30), which is present in a number of phorbol-12-myristate-13-acetate (PMA)-inducible promoters and confers inducibility to heterologous promoters (28–31). Interestingly, a similar structure is also found at the –55 to the –61 region (*TGAGTCT*) and continues to extend a sequence with high similarity to the human c-fos SRE that is present at the position –169 to –124 (32). Within the c-fos SRE homology, it contains an essential sequence (*ACGTCA*) of the cyclic AMP (cAMP)-response element (CRE). This CRE is also called multiple response element due to the characteristic that can be induced by serum, TPA, and Forskolin (33). Meanwhile, it is able to combine with cAMP-TPA-induced binding protein, namely, CRE-binding protein (CREB) (34, 35). Furthermore, this region contains not only a CRE motif but also the upper half of the 14bp NF-IL6-binding sequence that is located at the -163 to -145 (36, 37). All of these elements are conserved between human and mouse genes (38).

Both of the AP-1 and CRE sites have a high degree of sequence similarity because they have only a difference on two nucleotides (39). Downstream of the c-fos SRE, the presence of a putative binding site is located on the nuclear factor (NF)-κB transcription factor between -75 and -64 (30), which plays a crucial role in immunoglobulin κ gene expression (40). In addition to the aforementioned important regulatory sites, RBP (also designated CBF1) is a negative regulator that can downregulate expression of the NF-κB responsive IL-6 gene. It binds within the interleukin response element and hinders NF-κB coactivation with C/EBP-β. Its repression depends on the presence and position of the RBP target site within the IL-6 promoter (41). The CpG oligonucleotides on human IL-6 gene transcription reveal another negative regulator, retinoblastoma control element (RCE), which can be dissociated from the 3′-RCE binding site to enable the engagement of the C/EBP-β enhancer (42).

## Trans-Acting Factors

There are many trans-regulators that have been specifically identified and incorporated into the corresponding cis-regulatory elements so far. This incorporation plays a vital role on controlling the sequence of inflammatory response.

## Nuclear Factor-κB

Nuclear factor-κB is a pleiotropic transcription factor, whose main function is to direct transcriptional activity of various promoters of pro-inflammatory cytokines, especially for triggering IL-6 gene (43, 44). By using electrophoretic mobility shift assays, one can clearly observe that NF-κB binds specifically to the wild-type IL-6 promoter but not to the mutants. Libermann and Baltimore reported that the NF-κB-binding site is essential for the response of the IL-6 promoter to IL-1 and to TNF-α (45). It is a heterodimer consisting of two subunits: p50 and p65. p65 is the most frequent component of activated NF-κB and combines with high affinity to the consensus DNA sequences 5-*GGG PuN NPy PyC C-3*′(p65/p50) or 5′-*GGG PuN PyP yCC*-3′(p65/c-Rel) leading to the activation of transcription (46). NF-κB complexes were found inactive in the cells without stimulation and inhibited by a class of proteins called IκBa. Phosphorylation of the IκB is extremely responsible for dissociation of the inactive NF-κB dimers, which makes the latter translocate into the nucleus and binds to the IL-6 DNA (47). Furthermore, Palaga et al. confirmed that NF-κB directly involves in the positive regulation IL-6 gene by Notch1 signal in activated macrophages (48).

There are two major NF-κB activating signal transduction pathways. The classical pathway, which is initiated by pro-inflammatory cytokines, such as TNF-α binds to specific receptor causing the sequential recruitment of various stimulation factors, which makes the rapid phosphorylation of IκBa and dissociation from the NF-κB (49, 50). Another signaling pathway is a much slower process mediated by the NF-κB-inducible kinase. It leads to the phosphorylation of IKKα and the dimerization of p52 subunit and then follows through the classical pathway (49–51). NF-κB significantly mediates the activation of the IL-6 gene by a variety of IL-6 inducers such as PMA, LPS, TNF-α, poly(IC), and human T lymphotropic virus type I (52, 53). Nonetheless, the function is cell specific including U-937 monocytic cells, HeLa cells, and Jurkat T cells (49, 54–57). Meanwhile these inducible enhancer elements probably contribute to IL-6 gene induction in a cell-specific manner (45, 58).

## NF-IL6 and Its C/EBP Familty

NF-IL6, also known as C/EBP-β, was identified in 1990 as it can bind to a 14-bp palindromic sequence (*ACATTGCACAATCT*) within an IL-1 responsive element in the human IL-6 gene (59, 60). NF-IL6 expresses at an undetectable level in normal tissues but is obviously induced following the stimulation with LPS, IL-1, TNF-α, or IL-6 (52). It regulates a variety of genes involved in inflammatory cytokine (36). NF-IL6 contains a potential leucine zipper structure, which is highly homologous to a liver-specific transcriptional factor (30, 61).

The activity of NF-IL6 is regulated by several ways through its phosphorylation. First is the Thr-235 phosphorylated residue, second is within the leucine zipper, and third is the cAMP-mediated phosphorylation that can be associated with nuclear translocation and gene transcription (62, 63). NF-IL6 also cooperates with other transcriptional factors, playing a synergistic role in regulating the IL-6 gene expression. For example, p65, a subunit of NF-κB, results in the synergistic effect although this synergistic function comes to work only when NF-IL6 binds to its binding site of IL-6 promoter region (64, 65). In human intestinal epithelial cells, C/EBP-β (NF-IL6) may regulate IL-6 production through MAPK pathway. For example, when MAPK inhibitor (PD-98059) was used, it markedly attenuated the IL-6 mRNA and protein level, and this suppression is paralleled with the concentrations of PD-98059. In addition, this inhibition could involve in other transcription factors that can be influenced by MAPK signal pathways including AP-1 and NF-κB (65, 66). Although C/EBP interacts with NF-κB synergistically, the function of C/EBP on the IL-6 gene expression is diminutive without the NF-κB (67). Furthermore, C/EBP homologous protein (CHOP) upregulates IL-6 promoter activity at the transcriptional level with a manner dependent on the leucine zipper domain (68). Moreover, in the human melanoma cell line A375, CHOP raises the IL-6 production without binding to its promoter but trapping protein(s) such as liver-enriched inhibitory protein, an isoform of NF-IL6, which would otherwise inhibit IL-6 transcription (69). Within C/EBP family members, C/EBPζ (C/EBP heterodimers) acts as a negative regulator of IL-6 expression in B cells. Ectopic expression of C/EBPζ inhibited C/EBPζ-dependent IL-6 expression from both the endogenous IL-6 gene and the IL-6 promoter-reporter, while others such as C/EBPα, β, δ, and ϵ reveal the capacity of conferring LPS-inducible IL-6 transcription to P388 B lymphoblast, which is murine B lymphoblastic cell line (70, 71).

## Activator Protein 1

The AP-1, which belongs to the class of basic leucine zipper transcription factors, has homodimeric and heterodimeric complexes. AP-1-binding sequence (5′*TGAG/CTCA*3′), also known as the TPA response element, mediates the regulatory transcription of target genes (72, 73). AP-1 can bind to the specific binding site of the IL-6 promoter, exerting a significant effect on the regulation of IL-6 gene expression (74, 75). Combination of AP-1 with other transcriptional factors such as NF-κB and cAMP may have a synergistic role in transcriptional regulation of IL-6 gene in thyroid FRTL-5 cells, and the synergistic effects of TSH and cAMP on IL-6 secretion stimulated by IL-1 mainly involves the AP-1-binding site and an increase in the expression of its subunits: c-Fos and Fra-2 transcription factors (76). In IM9 cells, IL6-AP-1 is the most important cis-regulatory combination compared to other IL-6 promoter region: IL6–NF-κB, IL6-C/EBP, and IL6-CREB (77). However, there is a controversial observation on regulatory sites using IL-6 autocrine human prostate cancer cells. Xiao et al. showed that the cooperation between NF-κB and C/EBP-b is important for constitutively activated IL-6 promoter activity, while another study implicated the cooperation between NF-κB and AP-1 to be crucial (77, 78). Thus, the regulation of IL-6 gene expression is complex, and the AP-1 activation also works at a multilevel.

## Sp-1

Sp-1 is a ubiquitous transcription factor, which belongs to the Sp/XKLF family (79). Sp-1 binding site lies between the NF-IL6 and NF-κB enhancer, which is a G/C-rich sequence containing three repeats of the element *CCACC* in IL-6 gene (80). It is considered as an important bridge in binding between NF-κB and C/EBP isoforms in the IL-6 promoter (80). Sp-1 and NF-κB were demonstrated to have a positive cooperation in regulation of the human immunodeficiency virus promoter (81). In addition, Sp-1 may facilitate these interactions in IL-6 promoter for rapid response to inflammatory stimuli (82–84).

## Interferon Regulatory Factor (IRF)

Interferon regulatory factor could be one of other regulatory factors. By using the transient transfection of the chloramphenicol acetyltransferase (CAT) reporter gene linked to the IL-6 promoter to analyze the function of the 5′-flanking region of the IL-6 gene, it has demonstrated that IRF-binding site at position -267 to -254 is essential for induction of IL-6 gene expression following stimulation by IFN-γ. Transient transfection assays in HeLa cells demonstrated that the co-operation between IRF-1 and NF-κB at a low constitutive level is required for the comprehensive transcriptional activation of the IL-6 promoter directing CAT expression (85). IRF7 positively regulates IL-6 gene expression *via* enhancing IL-6 mRNA stability (86).

## Estrogen Receptor, Androgen Receptor, and Glucocorticoid

Apart from the positive transcriptional factors, some are also potent repressors involving in IL-6 gene expression. Steroid hormones, important regulators of physiological homeostasis, play a role on endogenous IL-6 expression.

Glucocorticoid classical function is mostly based on binding of a glucocorticoid receptor (GR) dimer to GREs in the regulatory regions of target genes including IL-6 (87). GRα, the pivotal subunit of GR, can repress pro-inflammatory gene by directly binding to a negative GRE, which involves the interactions between GRα and other transcription factors, particularly AP-1 and NF-κB (88, 89). P65 interacts with the GR and leads to mutual transcriptional antagonism in various studies *in vitro*. The interaction involves the DNA-binding domain of GR and the Rel homology domain of p65 (90). However, the exact mechanism is still controversial. It has been evident that p65 subunit of NF-κB and GR are physically interact. Physiological antagonism between two cytokines is based on a mutual transcriptional antagonism. On the other hand, others considered that NF-κB dissociation from DNA is not a requirement (87). Glucocorticoids may also repress NF-κB activity through induction of the NF-κB inhibitor IκB. Moreover, GR-mediated trans-repression is also through the direct protein–protein interactions between GRα and the c-Jun subunits (91).

Estrogens employ estrogen receptor to negatively regulate IL-6 gene expression *via* inhibition of the DNA-binding activities of the transcription factors NF-IL6 and NF-κB, as well as disruption of NF-κB transactivation (92, 93). Androgens also inhibit IL-6 gene expression *via* NF-κB. Using a prostate cancer cell line, dihydrotestosterone confirmed that it requires the androgen receptor to inhibit IL-6 gene promoter (94).

## Single-Nucleotide Polymorphism (SNP)

Polymorphisms in the promoter of IL-6 gene can result in interindividual variation in transcription and expression influencing an individual’s susceptibility to a diverse range of diseases (95). SNPs also play an important role in IL-6 gene expression that is related to common DNA sequence variations among individuals and associated to several human diseases. Up-to-date, four major polymorphisms of IL-6 have been identified where its polymorphism site is located at positions -572 G/C, -597 G/A, -1363 G/T, and -2954 G/C. These sites have a cooperative impact on the IL-6 gene transcription (96–98). A single nucleotide change from G to C at position -174 in the IL-6 promoter influences its transcription rate and is related to several diseases such as polycystic ovary syndrome (98–100). The – 174G/C SNP maps to a negative regulatory domain (−225 to −164), which approaches the CRE. Moreover, it is contained within a sequence bearing partial nucleotide homology with the Smad4-binding element and the “C” allele may bind Smad4 more effectively and thereby inhibit IL-6 transcription (100).

## Conclusion

Interleukin-6 acts as either a pro-inflammatory cytokine or an anti-inflammatory cytokine. Its role ranges from stimulating immune response, fighting infection, and responding to specific microbial molecules to changing the body’s temperature set-point, stimulating osteoclast formation, assisting hybridoma growth, as well as affecting muscle contraction. Both its upstream and downstream signaling pathways differ obviously between myocytes and macrophages, which involve in several pivotal protein molecules. IL-6 triggers its receptors CD130 and CD126 proteins to form a complex, thus initiating a signal transduction cascade through certain transcription factors like JAKs and STATs. Although IL-6 and its receptor are well acknowledged as a potential and vital target for the treatment of many diseases, there is still a large gap between cognization and utilization. Targeting the specific downstream molecular pathway may have the better therapeutic efficacy on inflammatory and autoimmune diseases.

## Author Contributions

YL wrote draft of MS and SZ edited and revised MS.

## Conflict of Interest Statement

The authors declare that the research was conducted in the absence of any commercial or financial relationships that could be construed as a potential conflict of interest.
